# Fostering the inclusion of teen fathers in adolescent health interventions: a call for structured and gender-inclusive programming

**DOI:** 10.1097/MS9.0000000000005241

**Published:** 2026-06-26

**Authors:** Yves Gashugi, Uwizera Marthe

**Affiliations:** a Global Health, University of Global Health Equity, Butaro, Rwanda; b Office of the Executive Director, Rwanda Psychological Society, Kigali, Rwanda; c Medicine and Pharmacy, University of Rwanda, Kigali, Rwanda

**Keywords:** adolescent health, adolescent pregnancy, father involvement, gender inclusion, teen fathers

## Abstract

Adolescent pregnancy remains a major global public health challenge, with significant consequences for health, education, and socioeconomic outcomes, particularly in low- and middle-income countries. While existing interventions have rightly prioritized adolescent mothers due to their biological and social vulnerabilities, this focus has inadvertently led to the systematic exclusion of teen fathers from adolescent health programming. Emerging evidence highlights that adolescent fathers play a critical role in maternal well-being, child development, and their own psychosocial outcomes. However, structural, cultural, and institutional barriers continue to limit their engagement. This commentary argues that adolescent parenthood is inherently relational and that excluding teen fathers undermines the effectiveness of current interventions. It calls for structured, gender-inclusive approaches that integrate teen fathers into adolescent health systems, promote shared responsibility, and strengthen outcomes for young families.

## Introduction

Adolescent pregnancy remains a significant global public health concern with far-reaching implications for the health, education, and socioeconomic trajectories of young people and their children^[^1^]^. Each year, an estimated 21 million girls aged 15–19 become pregnant globally, with approximately 95% of these births occurring in low- and middle-income countries, underscoring persistent global inequities in adolescent health outcomes^[^2^]^. It is also associated with severe health risks, contributing to several maternal deaths and approximately a million infant deaths annually, while significantly limiting educational attainment, increasing vulnerability to poverty, and reinforcing intergenerational cycles of inequality^[^3^]^.

Across diverse settings, considerable progress has been made in designing and implementing interventions that prioritize adolescent mothers, appropriately reflecting their heightened biological vulnerability during pregnancy and childbirth, as well as the disproportionate social and economic burdens they often experience^[^4,5^]^. These efforts, ranging from maternal health services to various supports such as school reintegration and psychosocial support, have rightly positioned adolescent girls as central beneficiaries of adolescent health programming^[^6,7^]^.

However, this necessary and justified focus has inadvertently contributed to a critical and persistent gap in both policy and practice: teen fathers remain largely invisible within adolescent health interventions. While adolescent motherhood is well documented and supported through structured programs, adolescent fatherhood is frequently overlooked, underexplored in research, and rarely addressed through targeted programming. This absence reflects not only a gap in service delivery but also a deeper systemic assumption that the responsibility for adolescent pregnancy and parenting rests primarily with girls.

Emerging global evidence suggests that adolescent fathers are not peripheral actors but important participants in child development, family dynamics, and long-term psychosocial outcomes^[^8^]^. Studies indicate that adolescent fatherhood is associated with both vulnerabilities, such as disrupted education, economic instability, and psychological distress, and potential positive trajectories, including increased responsibility, identity development, and reduced engagement in risky behaviors^[^9^]^. Yet, despite this evidence, structured interventions specifically designed for teen fathers remain limited, fragmented, or absent in most adolescent health systems.

This commentary argues that adolescent parenthood is inherently relational. Excluding teen fathers from intervention design weakens the effectiveness of existing programs by neglecting the shared social, emotional, and behavioral dynamics that influence maternal and child outcomes^[^10^]^. It also limits opportunities for positive male engagement, co-responsibility, and healthy identity formation during a critical developmental stage^[^11^]^. Therefore, the continued marginalization of teen fathers represents a significant blind spot in adolescent health programming, underscoring the urgent need for structured, gender-inclusive approaches that meaningfully integrate them into adolescent health interventions.

## The gendered design of adolescent health interventions

Adolescent health interventions addressing early pregnancy have largely been designed through a maternal lens, positioning girls as the primary, often sole, recipients of care, support, and accountability^[^12^]^. While this focus is justified, given the biological and social vulnerabilities faced by adolescent mothers, it has resulted in programs that are predominantly mother-centered^[^13^]^, with minimal consideration of adolescent fathers. Interventions such as sexual and reproductive health education, maternal health services, and psychosocial support initiatives tend to prioritize girls, reinforcing the implicit assumption that adolescent pregnancy is primarily a female responsibility^[^14^]^.

This gendered orientation is further embedded within the structure of health and social systems^[^15^]^. Antenatal and postnatal care services are typically organized around maternal and child health frameworks that do not actively include or engage adolescent boys. Similarly, school reintegration programs and social protection interventions often focus on supporting young mothers while overlooking the educational and psychosocial needs of young fathers. These patterns contribute to a gendered responsibility imbalance, where the burden of care, accountability, and social consequences is disproportionately placed on girls, while boys remain marginal to intervention efforts. Such exclusion not only reinforces inequitable gender norms but also weakens the effectiveness of adolescent health interventions by failing to address parenthood as a shared responsibility.

## Understanding teen fathers and the case for their inclusion

Teen fathers represent a diverse yet often overlooked population situated at a critical developmental transition between adolescence and emerging adulthood, a period marked by identity formation, educational pathways, and psychosocial maturation^[^16^]^. Assuming fatherhood during this stage frequently intersects with experiences of denial, fear, and stigma, compounded by social expectations to provide despite limited economic and emotional capacity^[^17^]^. In many contexts, adolescent fathers face pressure to conform to traditional provider roles without the resources or guidance to do so, leading to frustration, withdrawal, or disengagement. Importantly, such disengagement is often less a reflection of unwillingness and more a consequence of exclusion from support systems that are not designed to include them^[^18^]^. Contrary to dominant stereotypes, evidence suggests that many teen fathers express a desire to be involved, form emotional attachments to their children, and navigate fatherhood responsibly^[^19^]^, but lack structured opportunities, mentorship, and institutional recognition.

Recognizing and including teen fathers within adolescent health interventions is, therefore, not only a matter of equity but also a strategic imperative for improving outcomes. Their engagement has been associated with improved maternal well-being through shared emotional and practical support, as well as better child health and developmental outcomes, including cognitive and socio-emotional growth^[^20^]^. For the fathers themselves, inclusion can foster positive identity formation, reduce engagement in risky behaviors, and promote a sense of responsibility and purpose during a formative life stage. At the systems level, integrating teen fathers strengthens the effectiveness of existing interventions by addressing adolescent parenthood as a shared experience rather than an individual burden. Ultimately, fostering their inclusion can contribute to more balanced gender norms, enhance co-responsibility, and advance comprehensive, sustainable adolescent health programming.

## Barriers to inclusion

### Cultural norms

In many contexts, adolescent pregnancy and parenting are culturally constructed as primarily a female responsibility^[^21^]^. This mother-centric framing is reinforced by social expectations that position girls as the main caregivers and moral bearers of reproductive outcomes, while boys are often viewed as secondary or peripheral^[^22^]^. As a result, adolescent fathers are rarely expected, or encouraged, to participate in caregiving roles, which limits both their visibility and engagement in health and social support systems.

### Institutional design

Most adolescent health services are structurally embedded within maternal and child health platforms, particularly antenatal and postnatal care services^[^7^]^. These settings are typically designed for female attendance, both physically and programmatically, with limited provisions for adolescent boys. This institutional design unintentionally excludes teen fathers from routine engagement, reinforcing the perception that these services are not intended for them and reducing opportunities for their involvement in reproductive and child health processes.

### Stigma and policy gaps

Teen fathers are frequently subjected to stigma, often being labeled as irresponsible, absent, or disengaged, regardless of their actual intentions or capacities^[^19^]^. This stigmatization discourages participation and further alienates them from available services. At the same time, many adolescent health policies lack explicit provisions for male involvement in adolescent pregnancy and parenting, resulting in a policy vacuum that fails to recognize or protect the role of young fathers within health systems.

### Lack of research and funding focus

There remains a significant gap in empirical research on adolescent fatherhood, particularly in comparison with the extensive literature on adolescent mothers^[^9^]^. This imbalance has led to limited evidence to guide program design, weak advocacy for targeted interventions, and insufficient allocation of funding. Without robust data and sustained investment, teen father inclusion remains a marginal consideration rather than a prioritized component of adolescent health programming.

## Call for action

Addressing the exclusion of teen fathers requires deliberate, structured, and system-wide action across adolescent health programming. Interventions should move beyond mother-centered approaches by adopting father-inclusive strategies that recognize adolescent parenthood as a shared responsibility. This includes integrating co-parenting or couple-based approaches within existing services and establishing male-friendly entry points, such as schools, youth centers, and community or faith-based platforms, to improve accessibility and engagement. In parallel, targeted psychosocial support is essential, including mental health services, identity-focused interventions, and peer mentorship programs that connect young fathers with positive role models and supportive networks to strengthen coping, responsibility, and healthy identity formation.

At the systems level, there is a need for governments and institutions to formally integrate teen fathers into adolescent health policies and frameworks, ensuring they are recognized as active participants rather than peripheral actors. Strengthening data systems to capture male involvement in adolescent pregnancy and parenting is also critical for evidence-informed planning, monitoring, and accountability. Additionally, capacity building for service providers is necessary to shift practice from blame-oriented approaches toward inclusive, strengths-based engagement with adolescent boys. These efforts should be complemented by expanded interdisciplinary research, bringing together public health, psychology, education, and social work to develop and evaluate context-sensitive and scalable models of father-inclusive interventions across diverse settings.

In conclusion, adolescent parenthood is inherently relational, and interventions that focus solely on adolescent mothers remain incomplete. While continued prioritization of adolescent girls is essential due to their heightened biological and social vulnerability, the systematic exclusion of teen fathers represents a critical gap that undermines the effectiveness of adolescent health programming. There is an urgent need to transition from mother-centered models to inclusive, system-oriented approaches that engage both parents. Such a shift will promote shared responsibility, strengthen maternal and child outcomes, and support adolescent boys in developing into responsible, engaged fathers who contribute positively to families and society.

## Data Availability

Not applicable.

## Generative AI & reflexivity statement

Generative artificial intelligence (e.g., ChatGPT, OpenAI) was used in a limited and supportive capacity during the preparation of this manuscript, primarily to enhance language clarity, refine structure, and improve the organization of ideas. The tool assisted in articulating sections of the commentary, including the framing of arguments and overall coherence. However, all substantive content, including the conceptualization of the topic, critical analysis, interpretation of evidence, and development of recommendations, was independently conceived and developed by the author. No AI tool was used to generate original data, fabricate references, or produce unverified scientific claims. All outputs were critically reviewed, validated against existing literature, and revised to ensure academic rigor, accuracy, and integrity.

As a researcher and practitioner engaged in adolescent health, mental health systems, and community-based work, the author brings both professional and contextual insight into the subject of adolescent parenthood and gender dynamics in health interventions. This positionality informed the recognition of systemic gaps, particularly the marginalization of teen fathers within intervention design. At the same time, reflexive practices – including critical self-awareness, engagement with existing literature, and sensitivity to diverse sociocultural contexts – were employed to ensure balanced interpretation and to avoid overgeneralization. The commentary is thus grounded in both evidence and reflective analysis, aiming to contribute constructively to global discourse on inclusive adolescent health programming.
